# Characterization of the expression and immunological impact of the transcriptional activator CREB in renal cell carcinoma

**DOI:** 10.1186/s12967-020-02544-0

**Published:** 2020-09-29

**Authors:** Michael Friedrich, Christine Stoehr, Simon Jasinski-Bergner, Arndt Hartmann, Sven Wach, Bernd Wullich, André Steven, Barbara Seliger

**Affiliations:** 1 Institute of Medical Immunology, Martin Luther University Halle-Wittenberg, Magdeburger Str. 2, 06110 Halle (Saale), Germany; 2 Institute of Pathology, Friedrich Alexander University Erlangen-Nuremberg, Erlangen, Germany; 3 Department of Urology and Pediatric Urology, University Hospital Erlangen, Friedrich Alexander University Erlangen-Nuremberg, Erlangen, Germany

**Keywords:** CREB, HLA-G, Renal cell carcinoma, Immune cell infiltration

## Abstract

**Background:**

The non-classical human leukocyte antigen (HLA)-G is a strong immunomodulatory molecule. Under physiological conditions, HLA-G induces immunological tolerance in immune privileged tissues, while under pathophysiological situations it contributes to immune escape mechanisms. Therefore, HLA-G could act as a potential immune checkpoint for future anti-cancer immunotherapies. Recent data suggest an aberrant expression of the cAMP response element binding protein (CREB) in clear cell renal cell carcinoma (ccRCC), which is correlated with tumor grade and stage. Furthermore, preliminary reports demonstrated a connection of CREB as a control variable of HLA-G transcription due to CREB binding sites in the HLA-G promoter region. This study investigates the interaction between CREB and HLA-G in different renal cell carcinoma (RCC) subtypes and its correlation to clinical parameters.

**Methods:**

The direct interaction of CREB with the HLA-G promoter was investigated by chromatin immunoprecipitation in RCC cell systems. Furthermore, the expression of CREB and HLA-G was determined by immunohistochemistry using a tissue microarray (TMA) consisting of 453 RCC samples of distinct subtypes. Staining results were assessed for correlations to clinical parameters as well as to the composition of the immune cell infiltrate.

**Results:**

There exists a distinct expression pattern of HLA-G and CREB in the three main RCC subtypes. HLA-G and CREB expression were the lowest in chromophobe RCC lesions. However, the clinical relevance of CREB and HLA-G expression differed. Unlike HLA-G, high levels of CREB expression were positively associated to the overall survival of RCC patients. A slightly, but significantly elevated number of tumor infiltrating regulatory T cells was observed in tumors of high CREB expression. Whether this small increase is of clinical relevance has to be further investigated.

**Conclusions:**

An interaction of CREB with the HLA-G promoter could be validated in RCC cell lines. Thus, for the first time the expression of CREB and its interaction with the HLA-G in human RCCs has been shown, which might be of clinical relevance.

## Background

Renal cell carcinoma (RCC) is the most common form of kidney cancer. It arises from the renal tubular epithelial cells and can be further histologically subclassified into clear cell RCC (ccRCC), papillary RCC (pRCC), chromophobe RCC (chRCC) and several rare subtypes [1–3]. Interestingly, the incidence of RCC varies worldwide with the highest occurrence in North America and the Czech Republic [4]. RCC risk factors include obesity, smoking, diabetes mellitus and hypertension, among others [5]. Unfortunately, around 25–30% of RCC patients are diagnosed at a locally advanced or even at a metastatic stage, which negatively contributes to further therapy options and success. Recent studies demonstrate a decreasing RCC mortality between 1992 and 2015 in the USA. This might be attributed to improved diagnostics, like advanced abdominal imaging and/or to changes in the prevalence of RCC risk factors [6], as well as a grown range of therapeutic options. Treatment of RCCs is multidisciplinary. Besides surgical resection and radiotherapy, numerous medical treatments for metastatic disease have been approved in the last years including VEGF and VEGF-R inhibitors, [7, 8] mTOR inhibitors as well as with immune checkpoint inhibitors (ICI) [9]. Furthermore, first approaches with adoptive cell therapy (ACT) for RCC patients are under investigation, but despite the advances seen in melanoma, the reproducible generation of RCC tumor infiltrating lymphocytes (TILs) has been challenging [9].

Interestingly, the response rate to immunotherapies strongly vary between RCC patients, suggesting that the local composition of the tumor microenvironment and an altered expression of immune modulatory molecules might be crucial factors. The latter include the expression of PD-L1 and of the non-classical human leukocyte antigens (HLA) class Ib HLA-G and HLA-E as well as the down regulation of the classical HLA class Ia molecules mediated by an impaired expression of antigen processing machinery (APM) components [10–13]. Furthermore, cytokines secreted by Th1, Th2 and Th17 cells can also promote the progression of RCC [14]. Recent studies identified several microRNAs (miRs) regulating CREB in RCCs including miR-22-3p, miR-26a-5p, miR-27a-3p, and miR-221-3p [15].

Due to alternative splicing, HLA-G exists as membrane bound and soluble protein isoforms [16]. Both isoforms contribute to the composition and immune modulatory functions of the local tumor microenvironment [12]. Under physiologic conditions, its expression is restricted to mainly immune-privileged tissues including the cornea, testis, and the chorion. In contrast, a pathological HLA-G expression was found in many solid and hematopoietic tumors with an inter-tumor and intratumoral heterogeneity [17]. Furthermore, high HLA-G expression levels were associated with immune tolerance and inhibition of anti-tumoral immune responses [18, 19]. This was mediated by binding of HLA-G to inhibitory lymphocyte receptors, the immunoglobulin-like transcript (ILT)2, ILT4 and to the killer immunoglobulin-like receptor KIR2DL4 present on NK cells and CTLs [20].

The frequency of HLA-G expression in RCC lesions has been extensively investigated. Using a tissue microarray (TMA) consisting of 453 RCC lesions and matched normal kidney epithelium, a membranous HLA-G expression was found in 49.9% and a cytoplasmic HLA-G expression in 38.1% of cases, but the staining intensity strongly varied. Furthermore, the HLA-G expression was associated with the tumor grade: WHO grade 3 tumors often exhibited a stronger HLA-G staining than lower grade tumors. While the NK cell and CD4^+^ T cell infiltration did not vary, a significant difference in CD3^+^ and CD8^+^ cytotoxic T cells between HLA-G^+^ and HLA-G^−^ RCC lesions was observed [21].

HLA-G expression could be regulated by transcriptional, epigenetic as well as post-transcriptional mechanisms [22]. In addition, an alternative pathway of HLA-G gene transactivation mediated by the cAMP-response element-binding protein (CREB) has been reported in the literature [23]. This transactivation by CREB is unusual and differs from gene activation of the classical HLA genes mediated by NF-κB, IRF1 and the class II transactivator (CIITA).

CREB is a 43 kDa transcription factor, which binds after phosphorylation to the cAMP responsive element (CRE), a sequence that is localized in several gene promoters. Indeed, CREB functions are implicated amongst others in the regulation of cell proliferation, apoptosis, cycle progression and metastasis [24]. Indeed, CREB knock down in RCC cell lines suppressed RCC proliferation and decreased their tumor formation in nude mice [25]. In RCC lesions and in RCC cell lines, an increased CREB expression could be demonstrated [26], which was associated with a better RCC patients’ survival [15]. RCC is highly promoted by the composition of the tumor microenvironment (TME). Mutations of the von Hippel-Lindau (VHL) gene are common in ccRCC.

In this study, the role of the transcriptional transactivator CREB on the gene expression of the RCC-relevant immune inhibitory molecule HLA-G was investigated at the molecular level in RCC cell lines and in a large cohort of RCC lesions by immunohistochemistry (IHC). CREB, HLA-G and HLA-E staining results were tested for associations to clinical parameters. In addition, tumor immune cell infiltrate composition was investigated for different levels of CREB expression.

## Methods

### Cell lines and cell culture

The HLA-G positive choriocarcinoma cell line JEG-3 was purchased from the American Type Culture Collection (ATCC, Manassas, USA) and a set of five established RCC cell lines derived from patients with RCC (MZ1257RC, MZ2861RC, MZ2862RC, MZ2733RC and MZ2905RC) have been applied. With the exception of JEG-3 cells, which were maintained in RPMI 1640 (Invitrogen), the cell lines were cultured in Dulbecco’s modified Eagles medium (DMEM, Invitrogen) supplemented with 10% (V/V) fetal bovine serum (FCS; PAA), 2 mM L-glutamine (Lonza) and 1% penicillin/streptomycin (V/V; PAA).

### Tissue microarray and immunohistochemistry

Details of tissue micro array (TMA) construction and composition were published previously [21]. In short, the TMA consisted of samples from 453 formalin-fixed, paraffin-embedded RCC tissues which had been reevaluated by two experienced pathologists with respect to RCC subtype and WHO grade as defined by the 2004 World Health Organization (WHO) classification (NLM ID:101240923). After pathological review, a representative area per tumor had been transferred to recipient paraffin blocks, each capable of holding up to sixty tissue punches.

Five µm sections from the resulting eight TMA blocks were stained by conventional immunohistochemistry. The expression of HLA-G, HLA-E, CREB and immune cell marker expression have already been studied earlier on this TMA [15, 21, 27] and details of the staining procedures have been already been published. The authors reused these data to study the correlations to CREB expression.

Staining was evaluated by an experienced uropathologist (AH) and categorized for HLA-G, HLA-E and CREB by staining intensity: 0 (negative), 1 (weak), 2 (moderate), 3 (strong) [27]. For immune cells, four high-power fields (i.e. × 400) were evaluated for the mean absolute number of CD3^+^, CD4^+^, CD8^+^, and FoxP3^+^ cells [21].

From 2008 all patients gave informed consent, while obtained the Ethic Commission in Erlangen waived the need for informed individual consent for samples before 2008. The study is based on the approvals of the Ethic Commissions of the University Hospital Erlangen (No. 3755) and was conducted according to the principles expressed in the Declaration of Helsinki.

### Chromatin immunoprecipitation (ChIP)

ChIP assays were performed using a kit (Pierce™ Agarose ChIP Kit) from ThermoFisher (Waltham, USA) according to the manufacturer's instructions. Briefly, 2 × 10^6^ cells/assay were cross-linked in a 1% formaldehyde solution for 10 min at room temperature and the reaction was terminated by glycine (final concentration: 125 mM). Nuclei of cross-linked cells were isolated and DNA fragmentation was achieved by Micrococcal Nuclease digest. Antibodies against CREB1 (48H2, CST) and IgG (ThermoFisher) as isotype control were employed for immunoprecipitation overnight at 4 °C. ChIP samples were washed, eluted and DNA was UV-cross linked with NaCl and treated with proteinase K. Purification of the DNA was achieved by a column-based approach. Isolated DNA was subjected to (qRT-) PCR analysis.

### Quantitative PCR

To calculate the percent of input results ChIP samples, DNA was analyzed by qRT-PCR using GoTaq® qPCR Master Mix (Promega). Obtained Ct values for ChIP samples were normalized to input levels. For all primer pairs, an annealing temperature of 61 °C was used. Oligo-nucleotides (sequence 5‘ to 3‘) applied for amplification were HLA-G-Pro1_fw (AGGAGCAGGAGGTGAGGAAA), HLA-G-Pro1_rv (CAAAGAACACCCAGCGAAGC), HLA-G-Pro2_fw (AGTGAGGGGCATTGTGACTG), HLA-G-Pro2_rv (TATGTTGCAACCAGGAGCCA), RRM2_Pro_fw (GGGTCTCACTATGTTGCCC), RRM2_Pro_rv (CCCAGCACTTTGGGAGGCC).

### Western blotting

Total protein was extracted from different cell lines using RIPA buffer (25 mM Tris–HCl pH 7.6, 150 mM NaCl, 1% NP-40, 1% sodium deoxycholate, 0.1% SDS + protease inhibitor cocktail) and protein concentration was determined employing the Pierce™ BCA Protein Assay Kit (ThermoFisher). For Western blot analysis, 25 µg of protein / sample was separated by SDS-PAGE and transferred onto a nitrocellulose membrane by semidry blot. For detection of proteins, the anti-HLA-G antibody MEM-G/9 (Novus Biologicals), the anti-CREB1 antibody 48H2 (CST) and the anti-β-actin mAb ab8227 (Abcam) were used, while a horseradish peroxidase conjugated goat anti-α-mouse/rabbit antibody (CST) was employed as a secondary antibody. Chemiluminescent blots were imaged by LAS-3000 Imaging System (Fuji).

### Statistical analyses

Statistical tests on immunohistochemical results were performed using IBM SPSS Statistics 21 and 24 (IBM Corporation). Two-sided exact Chi-square test (Pearson’s or Fisher’s two sided exact tests, as appropriate) or Kruskal Wallis test were applied to test for correlations between tumor characteristics and tumor marker expression, or immune cell infiltrate characteristics and tumor marker expression, respectively. Associations of staining intensity and overall survival were calculated by log rank test. Differences were regarded significant at *p* < 0.05.

## Results

**Fig. 1:**
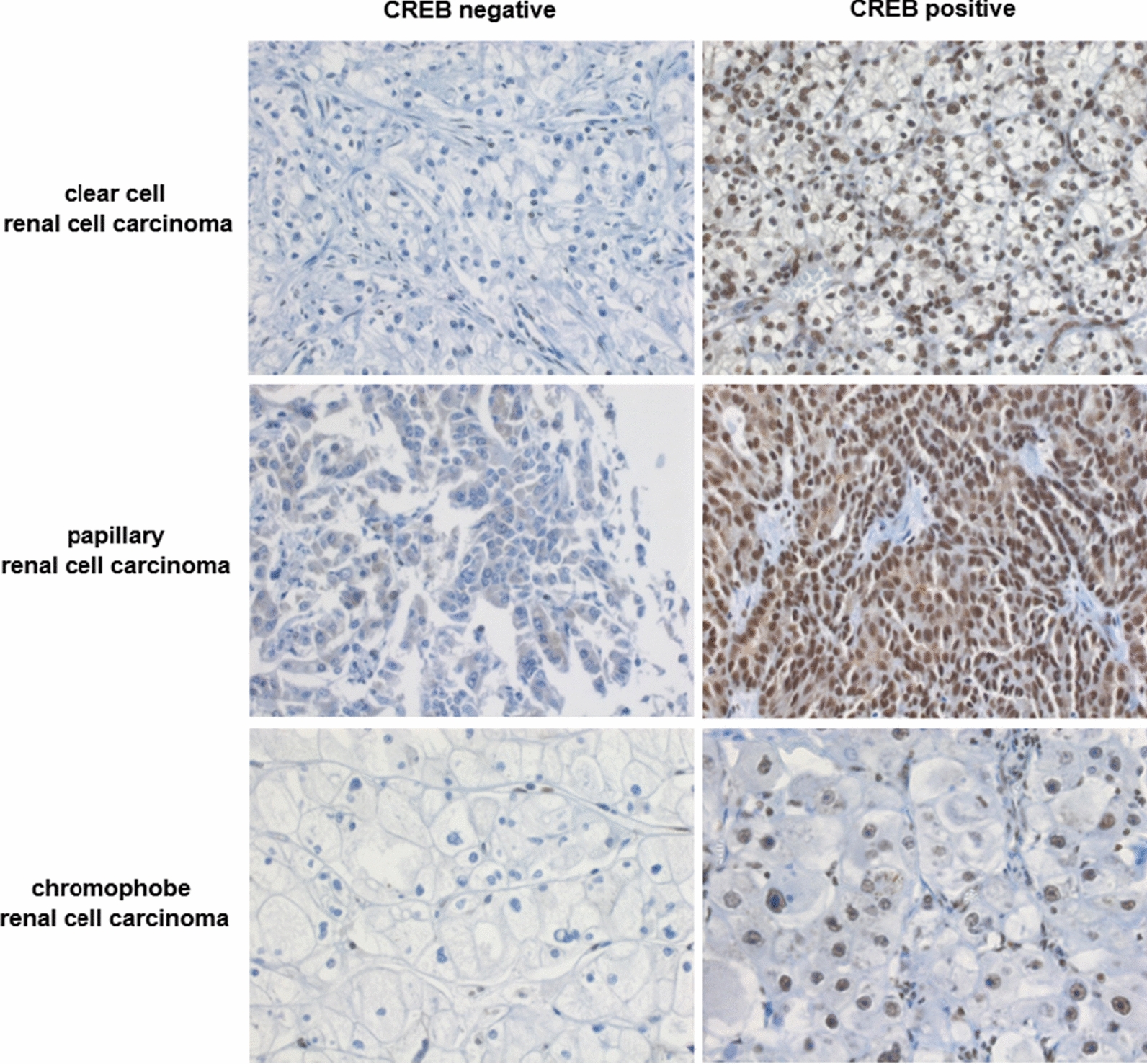
Representative CREB negative and positive specimen of the three most frequent RCC subtypes by immunohistochemistry (400-fold magnification)

In vitro experiments demonstrated a CREB-mediated induction of HLA-G transcription in extravillous cytotrophoblasts [23]. Previous studies analyzed the HLA-G and the HLA-E expression as well as the tumor infiltrating immune cell composition in a large cohort of RCC lesions using a TMA [20, 21, 27]. In the current study, the impact of the transcriptional activator CREB on the HLA-G expression was investigated in the RCC specimens. As recently shown, immunohistochemical staining of the RCC TMA with a pan-CREB mAb demonstrated a highly variable CREB expression in the RCC lesions ranging from negative, weak, medium to high expression [15] (Fig. 1).

**Fig. 2:**
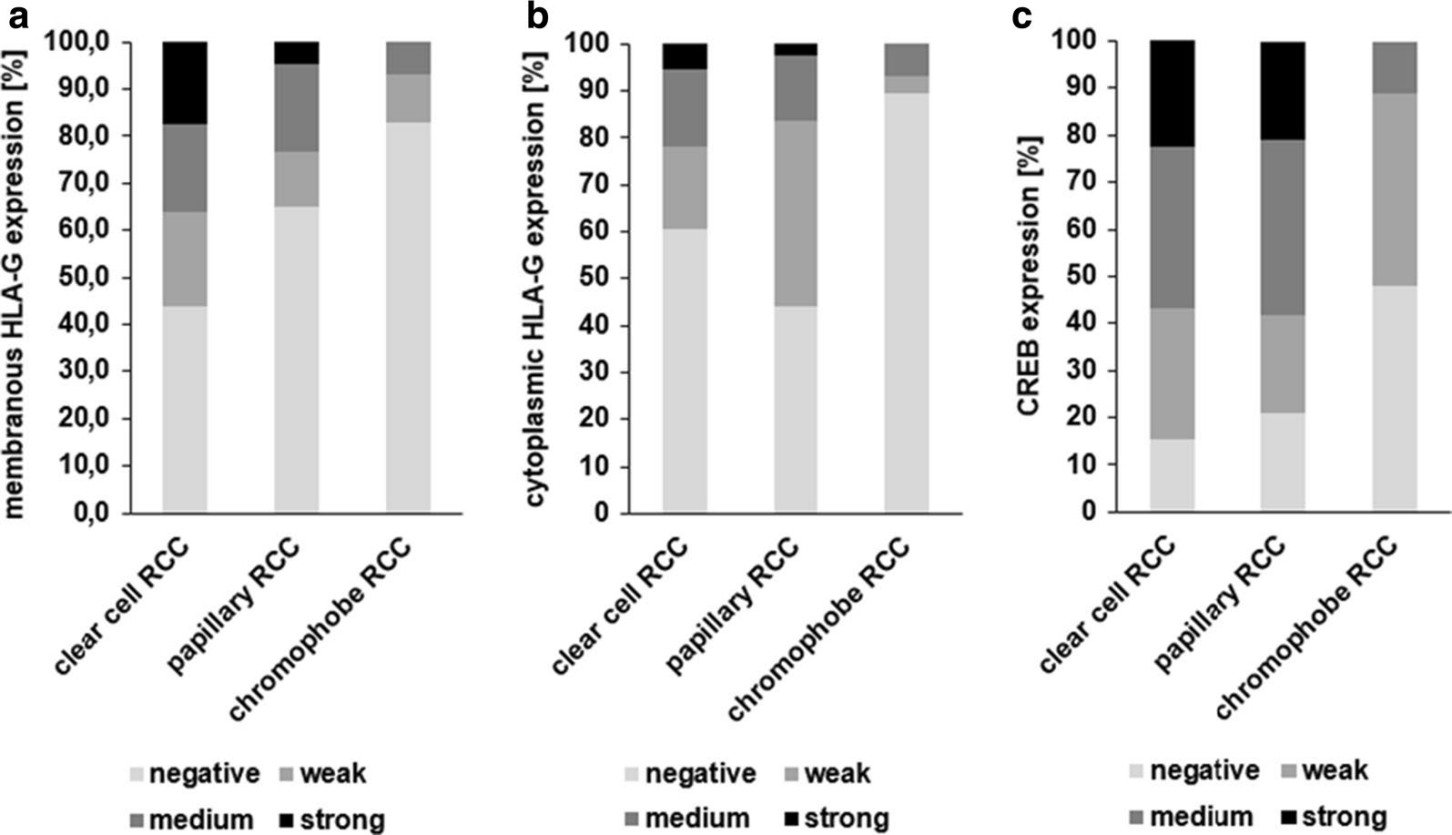
HLA-G and CREB expression levels in different RCC subtypes. The distribution of membranous HLA-G (A), cytoplasmic HLAG (B) and CREB (C) staining intensity varied significantly among clear cell (n = 333 and n = 314, respectively), papillary (n = 46 and n = 43, respectively) and chromophobe RCC (n = 29 and n = 27, respectively). p = 0.001 (membranous HLAG), p = 0.,002 (cytoplasmic HLAG), p < 0.001 (CREB))

The three major RCC subtypes ccRCC, pRCC, and chRCC, were separately analyzed for HLA-G and CREB expression by evaluating staining intensity distribution. The different RCC subtypes exhibit significantly distinct HLA-G and CREB expression (Fig. 2, p = 0.001 (membranous HLA-G), p = 0.002 (cytoplasmic HLAG), p < 0.001 (CREB)). The frequency of HLA-G expression was highest in ccRCCs (55.9% for membranous and 39.7% for cytoplasmic HLA-G) and pRCCs (39.8% for membranous and 58.7% for cytoplasmic HLA-G) when compared to chRCCs (16.2% for membranous and 10.3% for cytoplasmic HLA-G). Furthermore, the frequency of CREB expression was comparable to that of HLA-G expression. Medium and high CREB expressing tumors were more frequently found in ccRCCs (57.0%) and pRCCs (58.1) and rare in chRCC (11.1%).

**Fig. 3:**
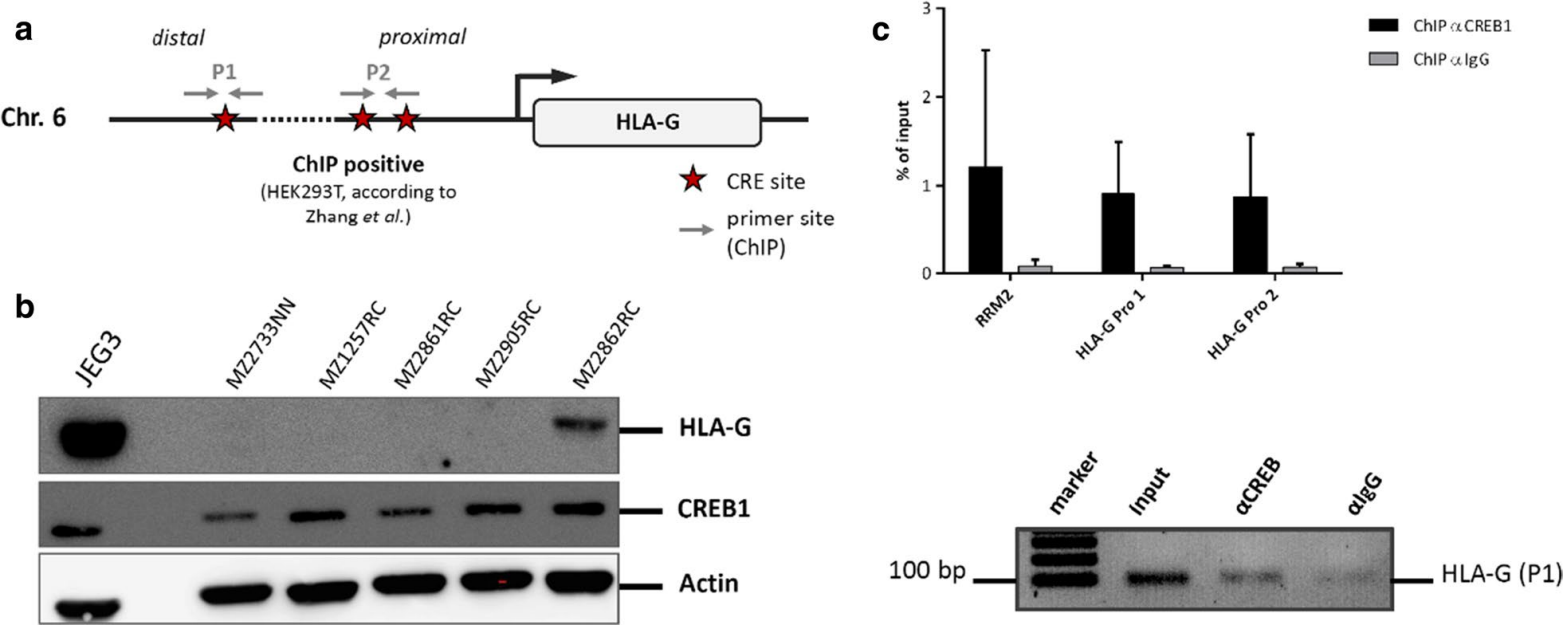
Analysis of the interaction of CREB with HLA-G. **a** In silico analysis of the HLA-G promoter sequence for predicted CREB binding sites. **b** The protein levels of HLA-G and of CREB were analyzed in RCC cell lines by Western blot. JEG-3 cells served as positive control. **c** The bar diagram shows the qPCR data expressed as means of three biological replicates of the chromatin immunoprecipitation. In comparison to the unspecific IgG control, the usage of anti-CREB antibody enriched HLA-G promoter regions as well as the internal positive control of the known CREB-bound RRM2 promoter sequence. The lower agarose gel exemplarily shows the enrichment of one HLA-G promoter fragment with the anti-CREB pull down in comparison to the isotype control of the antibody

Since a coordinated HLA-G and CREB expression was suggested by immunohistochemical staining, the molecular mechanisms were analyzed. In silico analyses revealed CREB-binding sites within the HLA-G promoter (Fig. 3a).To investigate whether the reported direct interaction of CREB with the HLA-G promoter sequence in extravillous cytotrophoblasts is also functional in the RCC cell system, ChIP was performed applying lysates from the RCC cell line MZ2862RC expressing both CREB and HLA-G (Fig. 3b). ChIP of the HLA-G promoter sequences including the CREB-binding sites using the anti-CREB antibody revealed a strong enrichment, which was almost as equal to the already known CREB regulated RRM2 promoter, which was used as positive control. This is shown by an exemplary agarose gel demonstrating an enrichment of the HLA-G promoter region with the anti-CREB mAb when compared to the respective isotype control (Fig. 3c). These results suggest that CREB is affecting the expression of HLA-G by binding to its promoter.

**Fig. 4:**
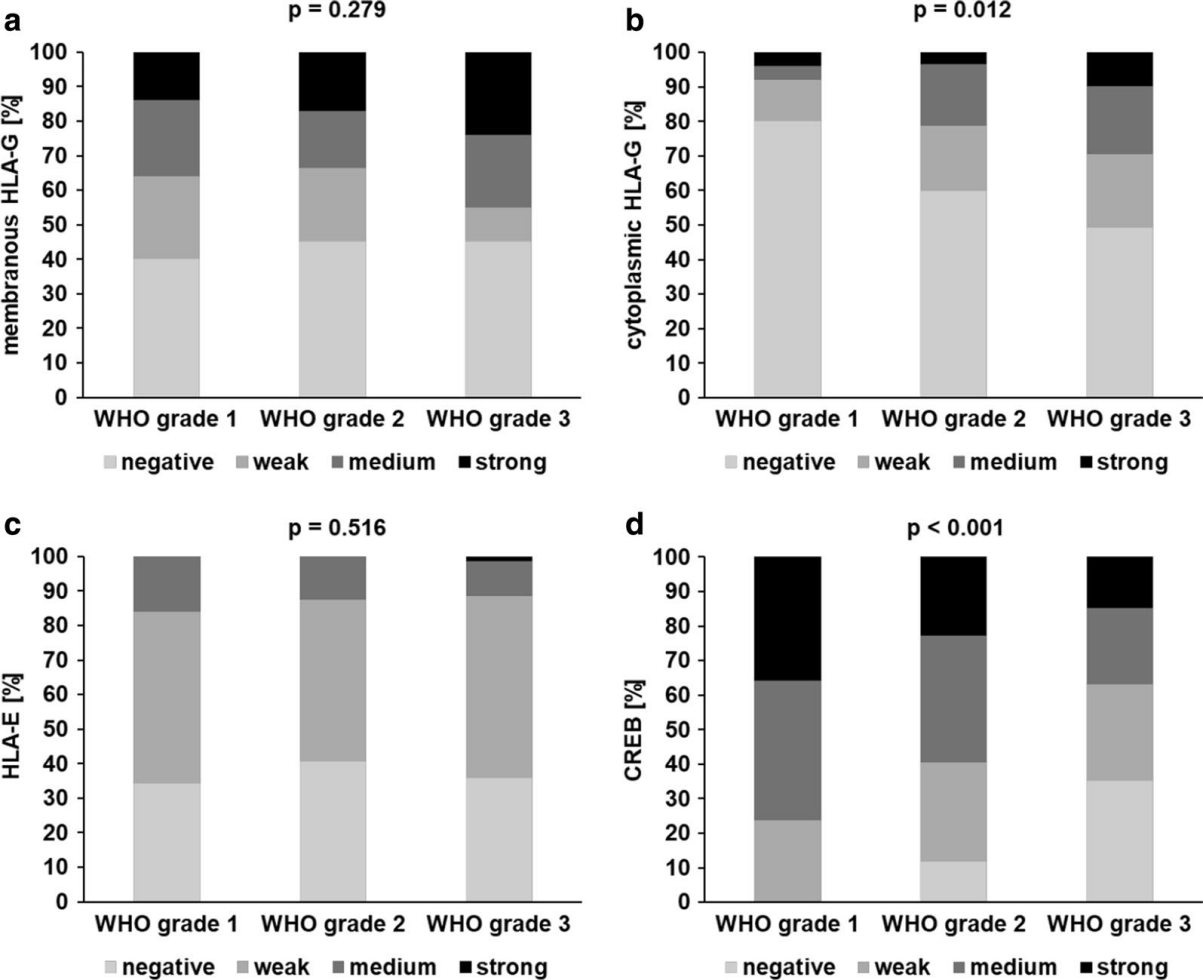
Correlation of HLA-G, HLA-E and CREB expression with grading of ccRCC. In ccRCC, staining intensity distribution among tumor grade categories varied for cytoplasmic HLA-G (B) and was inversely correlated for CREB (D), while there were no statistically significant differences for membranous HLA-G (A) or HLA-E (C)

To further address whether the coordinated expression of HLA-G, HLA-E and CREB has clinical relevance, their expression levels (staining intensity) were correlated to tumor grade. Analysis of HLA-E, which has no CREB-binding site in its promoter, served as control. Despite the heterogeneous expression in the different RCC subtypes only limited information about the tumor grade of the lesser frequent pRCC and missing validated scoring procedures for chRCC, the analyses were focused on ccRCC specimen (Fig. 4). While there was no statistically significant difference regarding HLA-E expression (p = 0.569) and membranous HLA-G expression (p = 0.279) among all tumor grades, cytoplasmic HLA-G expression was statistically significant associated with a higher tumor grade (p = 0.012). In contrast, CREB expression was statistically significant inversely correlated to tumor grade (p < 0.001): A lower CREB expression in ccRCCs was associated with a higher tumor grade as summarized in Table 1 suggesting that the HLA-G and CREB expression has independent effects on tumor grading.

**Table 1:** Expression (i. e. staining intensity) of CREB and HLA-G in ccRCC with respect to primary tumor WHO grade

ccRCC	WHO grade 1 n (%)	WHO grade 2 n (%)	WHO grade 3 n (%)
CREB			
Negative (0)	0 (0.0)	24 (50.0)	24 (50.0)
Weak (1)	10 (11.5)	58 (66.7)	19 (21.8)
Moderate (2)	17 (15.9)	75 (70.1)	15 (14.0)
Strong (3)	15 (21.1)	46 (64.8)	10 (14.1)
Cytoplasmic HLA-G			
Negative (0)	40 (19.9)	126 (62.7)	35 (17.4)
Weak (1)	6 (9.8)	40 (65.6)	15 (24.6)
Moderate (2)	2 (3.7)	38 (70.4)	14 (25.9)
Strong (3)	2 (12.5)	7 (43.8)	7 (43.8)
Membranous HLA-G			
Negative (0)	20 (13.6)	95 (64.6)	32 (21.8)
Weak (1)	12 (18.8)	45 (70.3)	7 (10.9)
Moderate (2)	11 (18.0)	35 (57.4)	15 (24.6))
Strong (3)	7 (11.7)	36 (60.0)	17 (28.3)

**Fig. 5:**
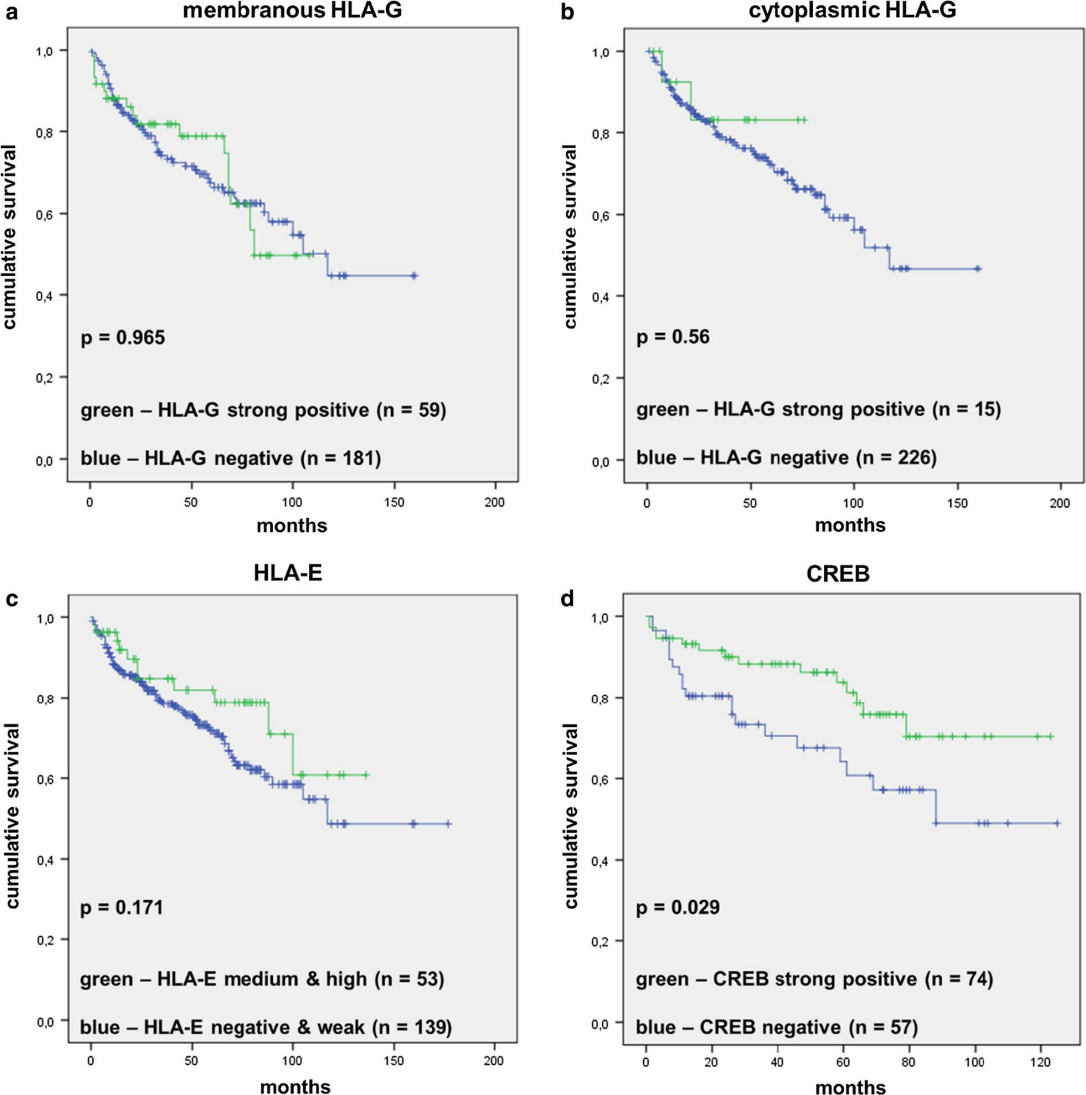
Correlation of HLA-G, HLA-E and CREB staining with ccRCC patients’ overall survival. HLA-G (A, B), HLA-E (C), and CREB (D) staining intensity was correlated with OS survival in RCC demstrating that only CREB expression was significantly (p = 0.029) associated to patients’ OS. Note: No survival data were available for HLA-E negative cases and only few data on strong cases. Therefore, for this marker, the staining categories “negative/weak” and “medium/strong” were compared

To analyze an association of HLA-G, HLA-E and CREB staining intensity on the overall survival of RCC patients, the respective non-expressing tumor specimen were compared to the strong positive specimens for HLA-G and CREB, respectively, and weak and moderate/strong specimens for HLA-E followed by the generation of Kaplan–Meier plots (Fig. 5). There was a statistically significant (p = 0.029) correlation of high levels of CREB expression with increased overall survival, while the survival of RCC patients did not statistically significant differ with the HLA-G expression (membranous or cytoplasmic) or HLA-E expression (p = 0.965, p = 0.56 and p = 0.216, respectively).

**Fig. 6:**
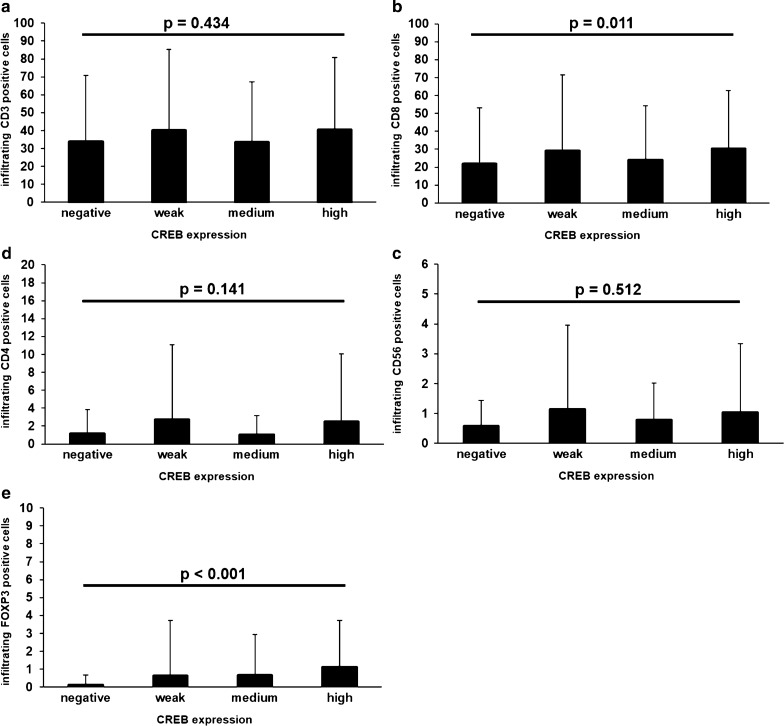
Analysis of the immune cell composition of the analyzed RCCs. Observed presence of different intra-tumoral immune cells among different categories of tumoral CREB expression (staining intensity). While the mean absolute numbers of four high power fields did not significantly vary for CD3+ (**a**), CD4+ (**c**) and CD56+ immune cells (p = 0.3, p = 0.11, p = 0.17, p = 0.12), the presence of FOXP3^+^ cell increased significantly with higher CREB expression (p = 0.0018), although the absolute cell number remains very low. CD8+ (**b**) expression also significantly vary (p = 0.011), but is hard to interprete when looking at the distribution of positive cells over the CREB staining categories

To address the question whether the CREB expression in RCCs is linked to alterations of the tumor infiltrating immune cell composition, the tumor infiltrating lymphocytes were analyzed and correlated to the CREB expression levels (staining intensity) of the RCC tumors (Fig. 6). There was no difference in the presence of the CD3 (p = 0.434) positive cells, which were predominantly CD8 positive CTL (p = 0.011) and to a lesser extent CD4 positive T helper cells (p = 0.141) in CREB^high^ or CREB^low^ RCC (Fig. 6). In addition, the frequency of CD56 positive cells (p = 0.512), including NK cells and NKT cells, was not associated to CREB expression levels. In contrast, the Treg frequency was statistically significant associated in the CREB expression (p < 0.0001): The higher the CREB expression of the tumor, the higher the amount of tumor infiltrating Tregs, although the mean absolute number of Tregs per high power field is rather low: non-CREB expressing tumors displayed 0.10 FoxP3^+^ immune cells per high power field in mean (median 0.00, std. deviation 0.56, minimum 0.00, maximum 4.25), while G3 tumors showed 1.10 FoxP3^+^ cells per high power field in mean (median 0.00, std. deviation 2.61, minimum 0.00, maximum 14.25).

## Discussion

The validation of reported in vitro studies regarding clinical relevant molecules in in situ and/or in in vivo systems is of great importance. The pathophysiologic HLA-G expression in solid und hematopoietic diseases is an important immune evasion strategy of tumors and linked to a certain composition of the TIL [21]. Therefore, the reported in vitro results of the suggested CREB-HLA-G interaction are for the first time analyzed in a clinical data set of human RCC specimen.

In the RCC specimen analyzed, the expression of HLA-G and HLA-E demonstrated no statistically significant association to overall survival of RCC patients. In contrast, the expression of the transcriptional activator CREB statistically significant correlated to the overall survival of RCC patients when comparing strong CREB positive to CREB negative tumors. This primarily contradictory finding might be explained by the pleiotropic functions of CREB or additional mechanisms involved in the regulation of the target gene or in patients’ survival.

The CREB expression of RCC tumors is also accompanied by an altered composition of TIL. Only the amount of FoxP3^+^ cells was statistically significant enhanced in CREB positive cells in comparison to CREB negative cells. FoxP3 expression is limited to Tregs [29] and known to suppress anti-tumoral immune responses. Although the total amount of infiltrating FoxP3^+^ Tregs was very low in the RCC samples examined, the number of TIL was associated to CREB expression levels with a concordant increase in CREB and HLA-G expression levels. This effect appears to be CREB-specific, since previous HLA-G expression studies in the same TMA did not reveal any HLA-G-dependent effect on FoxP3^+^ cells, but a strong statistically significant effect on CD3^+^ and CD8^+^ TIL [21].

Interestingly, Kim and co-authors identified CREB as a direct transcriptional activator of the FoxP3 gene expression in murine Tregs. This interaction of CREB with the respective sequence motif within the FoxP3 promoter region was furthermore dependent on its methylation status. Indeed, methylated DNA at the CREB binding site within the FoxP3 promoter prevented in vivo CREB binding [28]. These authors identified a TGACGTCA putative CREB site within the first intron of the FoxP3 gene, which was confirmed by the direct interaction and transcriptional activation of CREB at this site. Tregs are a subpopulation of CD4^+^ T cells and FoxP3 regulates their development in the thymus and maintenance in the periphery [29]. The number of tumor infiltrating Tregs within the strong CREB^+^ RCC tumors did not negatively contribute to the association of CREB expression with an increased overall survival and lower tumor grade.

In this study, the in vitro interaction of CREB and HLA-G in RCC cell lines was proven. This leads to the hypothesis whether CREB specific inhibitors like 666-15, which exerts an anti-cancer activity in mouse experiments both in vitro and in vivo [26], might be employed also for the RCC therapy. However, the weak in vivo effects of CREB on HLA-G expression as concluded from our data question any possible anti-cancer effects by CREB inhibition as a consequence of a down-regulated HLA-G transcription.

## Conclusions

The interaction of CREB and HLA-G was investigated in clinical human specimen and in different subtypes of RCC. In this study, both markers HLA-G and CREB showed an equal distributed expression independently of the RCC subtype. CREB expression in RCCs was inversely correlated to tumor grade, but positively correlated to overall survival and to the amount of tumor infiltrating Tregs. However, HLA-G expression exerts opposing functions and is linked to higher tumor grade. Therefore, further studies should investigate under which cellular conditions CREB physiologically induces HLA-G transcription and in which tissues and with which involvement of other regulatory mechanisms.

## Data Availability

The data and materials are available upon request.

## Abbreviations

ACT: Adoptive cell therapy
APM: Antigen processing machinery
ccRCC: Clear cell renal cell carcinoma
CD: Cluster of differentiation
ChIP: Chromatin immunoprecipitation
chRCC: Chromophobe renal cell carcinoma
CIITA: Class II transactivator protein
CTL: Cytotoxic T lymphocyte
CREB: CAMP-response element-binding protein
HLA: Human leukocyte antigen
ICI: Immune checkpoint inhibitors
ILT: Immunoglobulin-like transcript
mAb: Monoclonal antibody
miR: MicroRNA
NK: Natural killer cell
n.s.: Not significant
pRCC: Papillary renal cell carcinoma
TIL: Tumor infiltrating lymphocyte
TMA: Tissue microarray
TME: Tumor microenvironment
Treg: Regulatory T cell
VHL: Von Hippel Lindau
